# Chloral Hydrate in the Treatment of the Hyperemesis of Pregnancy

**Published:** 1886-03

**Authors:** D. D. Marr

**Affiliations:** Chesterton, Indiana


### DOMESTIC CORRESPONDENCE.

### CHLORAL HYDRATE IN THE TREATMENT OF THE HYPEREMESIS OF PREGNANCY.

CHESTERTON, INDIANA, February —, 1886.

TO THE EDITORS OF THE *Chicago Medical Journal and Examiner*.

*Gentlemen:*

Having noticed that chloral hydrate is not especially mentioned in medical literature as a remedy for alleviating that distressing, and at times uncontrollable, affection, the vomiting of pregnancy, it may not be amiss that I note my experience with this remedy.

Several years ago I was called to treat a patient affected with vomiting in pregnancy who had been previously treated by several physicians without relief.

I administered a rectal injection of chloral hydrate with the object of securing the much-needed sleep, which she had been unable to obtain for several nights.

I was very agreeably surprised, not only that I succeeded in my object, but that the vomiting immediately ceased, and did not return during that pregnancy. The patient rapidly recovered from her disagreeable condition, and was able to take a generous diet without the least discomfort.

Since this time I have invariably prescribed chloral hydrate injections for vomiting in pregnancy, always with the desired results.

Recently, in the treatment of a young woman married but a few months, I ordered this treatment, but she refused to disturb her usual rectal toilet, and the remedy was administered *per os*, with the same results as *per rectum*. Yet I prefer the latter method, and believe it is more certain in procuring relief, for very obvious reasons.

I hope that my fellow practitioners who have not used this remedy will submit it to trial, and I am sure their patients affected with vomiting in pregnancy will be better enabled to retain their morning meal.

Why this well-known drug controls this affection I will not say, but am content with the satisfaction of knowing that it will do so. D. D. MARR, M. D.

